# The role of extracellular DNA in *Neisseria* biofilms

**DOI:** 10.1128/jb.00314-26

**Published:** 2026-08-06

**Authors:** Jolyn Pan, Adele Williamson

**Affiliations:** 1 School of Science, University of Waikato 110948, Hamilton, New Zealand; Dartmouth College Geisel School of Medicine, Hanover, New Hampshire, USA

**Keywords:** biofilm, *Neisseria*, extracellular DNA, commensal, pathogen, gonorrhea, meningitis

## Abstract

The genus *Neisseria* includes two human pathogens, *Neisseria gonorrhoeae* and *Neisseria meningitidis*, in addition to commensal species that also inhabit human and animal hosts. One of the features that occurs during neisserial host colonization is biofilm formation. In other bacteria, biofilm formation enables persistence as well as evasion of the host immune system. Extracellular DNA (eDNA) forms a major component of *Neisseria* biofilms and contributes to initial steps in their formation, as well as the structure and stability of the biofilm extracellular matrix. Here, we review the potential roles and sources of eDNA in *Neisseria* biofilms and its relevance to both pathogenic and commensal *Neisseria* spp. We also highlight the paucity in research on biofilm formation by commensal *Neisseria* and hope this will stimulate further research on the role of eDNA in nonpathogenic *Neisseria*.

## INTRODUCTION

The genus *Neisseria* are a group of gram-negative β-proteobacteria that colonize different mucosal surfaces in animals and are characterized by either a diplococci or rod morphology and lack of flagella (1). Of the 38 species identified to date, 15 have been isolated from humans forming part of the normal human microbiota, while two, *Neisseria meningitidis* and *Neisseria gonorrhoeae*, are classified as human pathogens. *N. meningitidis* is an opportunistic facultative human pathogen that can cause meningitis and sepsis, while *N. gonorrhoeae* is an obligate human pathogen that infects the mucosal cells of the reproductive tract, causing the sexually transmitted infection (STI), gonorrhea. *N. gonorrhoeae* also readily infects other locations, including the oropharynx and the rectum; in studies of heterosexual patients, ~30% of patients with a reproductive tract gonococcal infection also have oropharyngeal gonorrhea (2), while oral and anal gonococcal infections are particularly prevalent in men who have sex with men (MSM) populations (3). Both pathogenic *Neisseria* species are high priorities for treatment and surveillance due to the adverse health outcomes associated with infection. For meningococcal disease, these include high mortality rates or life-limiting disability (4), while gonorrhea infections can cause infertility or ectopic pregnancy in women (5). Like all *Neisseria*, *N. meningitidis* and *N. gonorrhoeae* are constitutively competent, enabling rapid acquisition of antibiotic resistance genes and leading to multidrug-resistant infections (6). In addition, both species can undergo extensive-phase and antigenic variation, which further allows them to evade the host immune response (7, 8).

Despite much *Neisseria* research being focused on pathogenic species, most of the human-colonizing members of this genus are commensal. These share a similar habitat to their disease-causing counterparts in the oropharynx and nasopharynx, and although there are reports of disease arising in immunocompromised individuals (Table 1), virulence is typically low (9–11). These commensal *Neisseria* are important for maintaining healthy interactions with other beneficial microbes by suppressing the growth of pathogenic *Neisseria* through resource competition (12–14), as well as direct mechanisms such as production of secondary metabolites (15) or DNA-mediated killing ascribed to different methylation patterns between commensals and their pathogenic counterparts (16).

**TABLE 1:** Characterized commensal *Neisseri*a spp. that colonize human mucosal surfaces^*a*^

Species	Habitat	Characteristics and potential infections
*Neisseria bacilliformis* (17)	Oropharynx and respiratory tract	Rod-shaped. Associated with endocarditis (18)
*Neisseria bergeri* (19)	Oropharynx	Not associated with disease
*Neisseria cinerea* (20)	Oropharynx and genitourinary tract	Reports of neonatal conjunctivitis obtained from the mother (21, 22)
*Neisseria elongata* (23) (subspecies *elongata, glycolytica,* and *nitroreducens*)	Nasopharynx and oropharynx	Rod-shaped. Associated with endocarditis and infected lung bulla (24)
*Neisseria lactamica* (25)	Nasopharynx	Same habitat and similar traits as *N. meningitidis*; potentially protective against *N. meningitidis*. Common in children and neonates, but infection rates decrease with age
*Neisseria mucosa* (26)	Nasopharynx and oropharynx	May be opportunistic in immunocompromised patients (i.e., endocarditis) (27–29). Is the most commonly reported invasive commensal *Neisseria* spp. (10)
*Neisseria oralis* (30)	Oropharynx	Associated with healthy gingival plaque (30)
*Neisseria polysaccharea* (31)	Nasopharynx and oropharynx	Able to produce extracellular polysaccharides from sucrose. No reports of disease (10)
*Neisseria skkuensis* (32)	Oropharynx	Associated with infective endocarditis (33)
*Neisseria subflava* (34)	Upper respiratory tract and genitourinary tract	May be opportunistic in immunocompromised patients (i.e., endocarditis, meningitis, and septicemia), but these are rare (35–37)

^*a*^ Nomenclature based on reference 38.

A common feature of pathogenic and some commensal *Neisseria* is their ability to form biofilms. Such biofilms offer multiple benefits to the cells enclosed within them, such as facilitating molecular communication and substrate exchange between neighboring cells, and possible exchange of genes encoding antibiotic resistance (39). Unlike most bacteria, *Neisseria* spp. lack the genes to produce exopolysaccharides, which is a major component of the extracellular matrix (ECM) in most bacterial biofilms (40). Instead, *Neisseria* biofilms form primarily from the secretion of other substrates including outer membrane vesicles and extracellular DNA (eDNA) (41). In this report, we review the functional role of eDNA in *Neisseria* biofilms with a focus on its potential origin(s) and incorporation. Most of our knowledge about *Neisseria* biofilms comes from the two pathogenic species, which highlights the paucity of knowledge on biofilm formation by commensal *Neisseria* spp.

## BIOFILM FORMATION BY *NEISSERIA*

The importance of biofilm formation in *N. meningitidis* was initially demonstrated where formation of biofilms *in vitro* enhanced their resistance to penicillin (42). Initially only unencapsulated strains of *N. meningitidis* were shown to form biofilms using *in vitro* assays; however, a few capsulated strains isolated from patient samples have since been shown to generate biofilms on airway epithelial cells *in vitro* (42, 43). The finding that unencapsulated strains of *N. meningitidis* were more likely to form biofilms was further supported by transcriptomic analysis, which showed a downregulation of capsule synthesis genes during biofilm formation (44, 45). This is relevant as capsulated *N. meningitidis* strains are typically more invasive, often leading to disease; however, capsulation can be lost through phase variation, indicating that modulation of capsule expression and subsequent biofilm formation in *N. meningitidis* relies on environmental cues to ensure efficient attachment and colonization before invasion into host cells (46).

*N. gonorrhoeae* has similarly been shown to form biofilms in both flow chambers and on primary urethral epithelial and cervical cells (47), as well as *in vivo* in cervical biopsy samples (48), and it has been shown to colonize intrauterine contraceptive devices as a minor part of a mixed biofilm with *Staphylococcus aureus* and *Candida albicans* (49). While highlighting the importance of *N. gonorrhoeae* biofilms in pathogenesis in women, there is little evidence of *N. gonorrhoeae* biofilm formation in male patients, and since many gonococcal infections in women are asymptomatic, this may indicate a role for biofilms in evading the host immune response (50).

Less is known about biofilm formation in commensal *Neisseria* compared to their pathogenic counterparts. Several *Neisseria* that colonize the human oral cavity including *Neisseria bacilliformis* and *Neisseria mucosa* form mixed-species biofilms with non-*Neisseria* microbes, which is implicated in the formation of gingival plaques (51, 52). These biofilms likely facilitate colonization by commensal *Neisseria* of their environment and survival against competitors; however mixed biofilms of commensal and pathogenic *Neisseria* strains have also been reported, for example, between *Neisseria lactamica* and *N. meningitidis,* which coexist in the nasopharyngeal tract (53).

## FUNCTION AND METABOLIC ENVIRONMENT OF BIOFILMS IN *NEISSERIA*

Bacterial biofilms often form in stressful environments where they increase bacterial survival and persistence (54, 55). By creating a physical barrier between the cells and the environment, biofilms protect bacteria from environmental stressors including oxidative stress, dehydration, shear forces and UV radiation, as well as host immune responses and external agents like antimicrobials (39). *N. meningitidis* cells inside a biofilm are more resistant to penicillin than their planktonic counterparts due to the depth that the antibiotic has to penetrate to reach cells at the biofilm center (42), and aggregation in *N. gonorrhoeae* has been linked to the upregulation of genes involved in antibiotic tolerance and observed tolerance to ciprofloxacin (56). This physical barrier also generates unique gradients of substrates, nutrients, and pH that make it more difficult for the host to specifically target the infection due to the variation in phenotypes, behaviors, and metabolic responses (56). Finally, biofilm formation aids in the physical attachment of pathogenic bacteria to their target host cells, which increases their chances of infection and invasion, and hence contributes to their pathogenicity and virulence (48, 57).

Consistent with its role in survival, transcriptional and proteomic profiling of gonococcal biofilm showed an upregulation of genes important for stress response and anaerobic respiration such as the nitric oxide reductase, *norB*, and the cytochrome c peroxidase, *ccp*, especially by cells in the biofilm base layers (56, 58, 59). Disruption of these genes in *N. gonorrhoeae* decreased biofilm biomass and thickness, suggesting that anaerobic respiration occurs in *N. gonorrhoeae* biofilms (56, 58, 59). More specifically, during these oxygen-limiting conditions, *N. gonorrhoeae* uses partial denitrification to reduce nitrite to nitrous oxide, a pathway that has also been demonstrated to occur in *N. meningitidis* (58). Through this nitrite reduction in biofilms, *N. gonorrhoeae* may break down host-produced nitrous products, which would subsequently dampen the host inflammatory and immune responses and result in asymptomatic infections (58, 60). The formation of biofilms by *N. gonorrhoeae* as an adaptation to oxidative stress is further supported by the inability of mutants deficient in some redox-related genes such as the oxidative stress regulatory protein, *oxyR*, and the glutathione reductase, *gor*, to form biofilms (58).

Other metabolic factors that affect the formation of *Neisseria* biofilms include the carbon sources available in the host environment. For example, L-lactate has been shown to reduce biofilm formation in *N. gonorrhoeae* and causes microcolony dispersal in *N. meningitidis* (61–63). Further, many L-lactate regulon genes have been shown to be downregulated during gonococcal biofilm formation, suggesting that it occurs under slow anaerobic respiration as opposed to the conditions of aerobic growth and high energy (L-lactate) consumption during biofilm dispersal (61, 63). Interestingly, proteins important in binding and transporting iron are downregulated during *N. gonorrhoeae* biofilm formation, which may also indicate reduced metabolic and growth rates during biofilm formation (59).

## THE ROLE OF EXTRACELLULAR DNA IN FORMATION AND INTEGRITY OF *NEISSERIA* BIOFILMS

Four major steps have been identified in bacterial biofilm formation: (i) reversible monolayer formation after bacteria-surface contact, (ii) formation of multilayered microcolonies and production of an ECM, (iii) three-dimensional biofilm formation, and finally (iv) cell dispersal (64, 65). In most bacteria, secreted exopolysaccharide is the primary ECM constituent (~50%–90% of the biofilm), where it aids with cell-cell and cell-surface adhesion, as well as protecting cells (54, 66). *Neisseria,* by contrast, lack the genes necessary for exopolysaccharide production; instead, neisserial biofilms comprise a combination of extracellular DNA (eDNA) and outer membrane vesicles. These outer membrane vesicles pinch off the cells to shape the matrix and stabilize the biofilm, and mutation of the *msbB* gene of *N. gonorrhoeae,* which causes decreased membrane blebbing, reduces its ability to form biofilms in flow cells (47, 48). The membranous biofilm matrix of *N. gonorrhoeae* also contains multiple water channels and pores that facilitate nutrient and substrate exchange (47). The structure of biofilms formed by *N. meningitidis,* by contrast, are highly strain-dependent, with some exhibiting very well-defined connection channels, while others seem to show no evidence of any defined connections (67).

The most notable feature of all neisserial biofilms, however, is the large quantity of eDNA present in the ECM. While eDNA only comprises a few percent of the ECM in most bacteria, in *Neisseria,* it is a major contributor to biofilm formation and integrity (41). eDNA appears to play a dual role in the ECM at different stages of meningococcal biofilm formation, first by supporting the initial attachment of the bacteria to the substratum during early biofilm stages when the levels of eDNA are relatively low and second by maintaining mechanical integrity during late stages when the levels of eDNA are higher (68).

**Fig 1:**
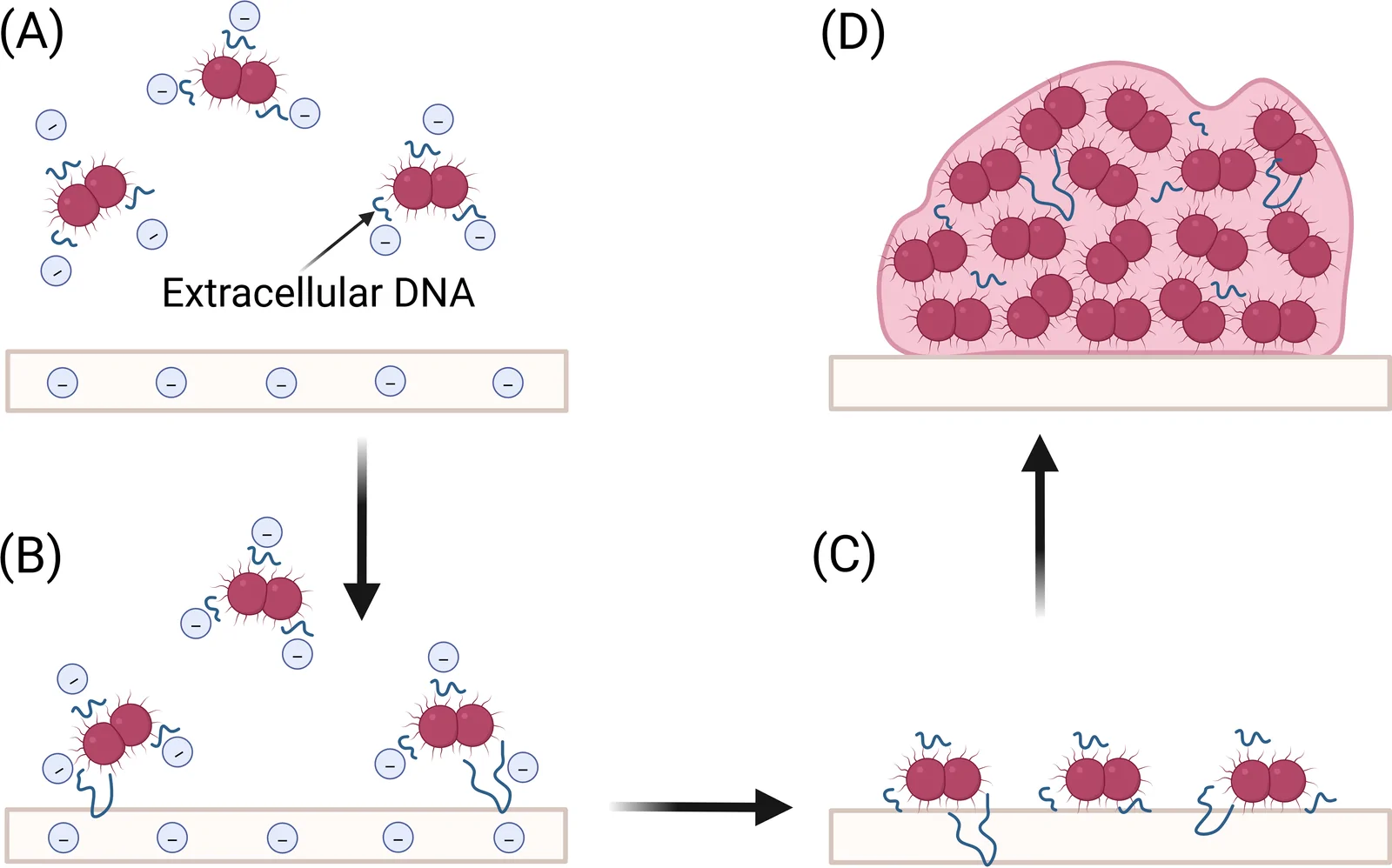
Role of extracellular DNA in bridging repulsive forces during biofilm formation initiation in *Neisseria*. (**A**) Weak repulsive forces exist between the bacteria and surface of interest. (**B**) High-molecular-weight extracellular DNA extend out and bridge the repulsive forces, facilitating close-range interactions between the bacteria and surface. (**C**) Reversible formation of a monolayer on the surface. (**D**) Cell replication and release of extracellular matrix leads to formation of mature, multilayered three-dimensional biofilms. Created in https://BioRender.com.

During the establishment of neisserial biofilm, eDNA aids adhesion of bacteria to the substrate. In general, attachment of cells to a surface involves both attractive long-range Liftshitz-van der Waal interactions and weak repulsive interactions that arise from the mutual net negative charges that exist on most cellular and abiotic surfaces (69, 70). eDNA can facilitate closer-range interactions by adsorbing to and extending out of cellular surfaces in long loop structures (Fig. 1). These structures are then able to interact with any acidic (electron-accepting) or basic (electron-donating) groups via close-range acid-base interactions (68, 69, 71, 72). It is likely that this initial eDNA-dependent bacterial attachment forms the monolayer that the *Neisseria* biofilm grows from, although this is an ongoing area of investigation.

Perhaps the best recognized function of eDNA in biofilm, however, is its role in maintaining the structure and stability of the biofilm matrix. After the formation of the monolayer on surfaces, the division of *Neisseria* cells is accompanied by different interactions between neighboring cells that allow them to be maintained as three-dimensional microcolonies. Here, the negatively charged eDNA likely acts as an adhesive network by interacting electrostatically with any positively charged surface-exposed matrix proteins of neighboring cells (67, 73, 74).

Single-stranded DNA appears to be more important for initial biofilm formation in *N. gonorrhoeae*, while double-stranded DNA makes up the majority of eDNA in mature gonococcal biofilms (75). Furthermore, recent work has shown that the length of eDNA governs its mobility within gonococcal colonies and hence its subsequent retention and incorporation into the ECM (76). The prevalence of eDNA for *N. meningitidis* biofilm formation differs between strains, which has given rise to the “settler/spreader” biofilm model. Here, eDNA-independent clonal complexes, or “spreaders”, form unstable, fragile biofilms that are more sensitive to shear forces but have higher transmission rates between different hosts due to their ease of detachment (53, 68). This includes the hypervirulent clonal complexes ST-8 and ST-11 that are less common among healthy carriers but are causes of invasive meningococcal disease (45). Conversely, eDNA-dependent clonal complexes or “settlers” form stronger, more robust biofilms that are quickly established and allow for more stable interactions and long-term colonization of the human host to occur (68). The combination of both “settler” and “spreader” phenotypes in a population allows *N. meningitidis* to effectively colonize and spread among different hosts over long periods of time, promoting its virulence and pathogenicity (45).

## NON-STRUCTURAL FUNCTIONS OF DNA IN *NEISSERIA* BIOFILMS

In addition to providing structural integrity, the large quantity of eDNA in *Neisseria* biofilms represents a pool of available DNA that may be used for other cellular processes. DNA provides a significant source of phosphate, nitrogen, and carbon, and recycling eDNA decreases the energy requirements for *de novo* nucleotide synthesis under stressful conditions (77, 78). Although not yet observed in *Neisseria* spp., the recent discovery that eDNA is produced during biofilm establishment in *Bacillus subtilis* before its global degradation in a spatiotemporally coordinated pulse has led to the conclusion that eDNA can act as a “metabolic reservoir” that can be used when nutrient availability decreases in later stages of growth (79). Alternately, it is possible that competent *Neisseria* may incorporate eDNA for genomic DNA repair. This theory is consistent with the observation that only eDNA with a specific DNA uptake sequence is internalized by *Neisseria* spp., guaranteeing that only closely related DNA suitable for homologous recombination repair pathways is acquired (80). Finally, eDNA in *Neisseria* biofilms provides a potential source for acquisition of novel genes from neighboring cells including new antibiotic resistance and virulence genes that may increase overall fitness and survival (81). Considering that *Neisseria* are naturally competent at all stages of growth, the use of eDNA for DNA repair and horizontal gene acquisition is highly likely.

Recent studies using fluorescently labeled DNA suggest an interplay between genetic transformation and biofilm formation in *N. gonorrhoeae* based on the length of eDNA present (76). Here, small fragments of DNA of around 300 bp were shown to diffuse freely through *N. gonorrhoeae* colonies and were likely taken up by the cells, whereas those exceeding 3,000 bp had hindered diffusion and were more likely to be slowly incorporated into the biofilm ECM (76). This is supported by the observation that DNA transformation and acquisition of multidrug resistance in *N. gonorrhoeae* is highly efficient during early biofilm formation (<24 h), which is where eDNA is predicted to contribute to *Neisseria* biofilm formation the most, but was strongly reduced after 24 h, independent of biofilm density (82). This feature is not specific to *Neisseria,* however, as higher transformation or conjugation rates have also been observed in *Escherichia coli* biofilms compared to planktonic cells (83), in mixed biofilms from dental plaque (84), and in *Streptococcus mutans* upon activation of proteins involved in competence (85–87). Taken together, these observations indicate that biofilms are highly important at facilitating horizontal gene transfer in general, including among *Neisseria* (69).

## SOURCES OF EXTRACELLULAR DNA IN *NEISSERIA* BIOFILMS

**Fig 2:**
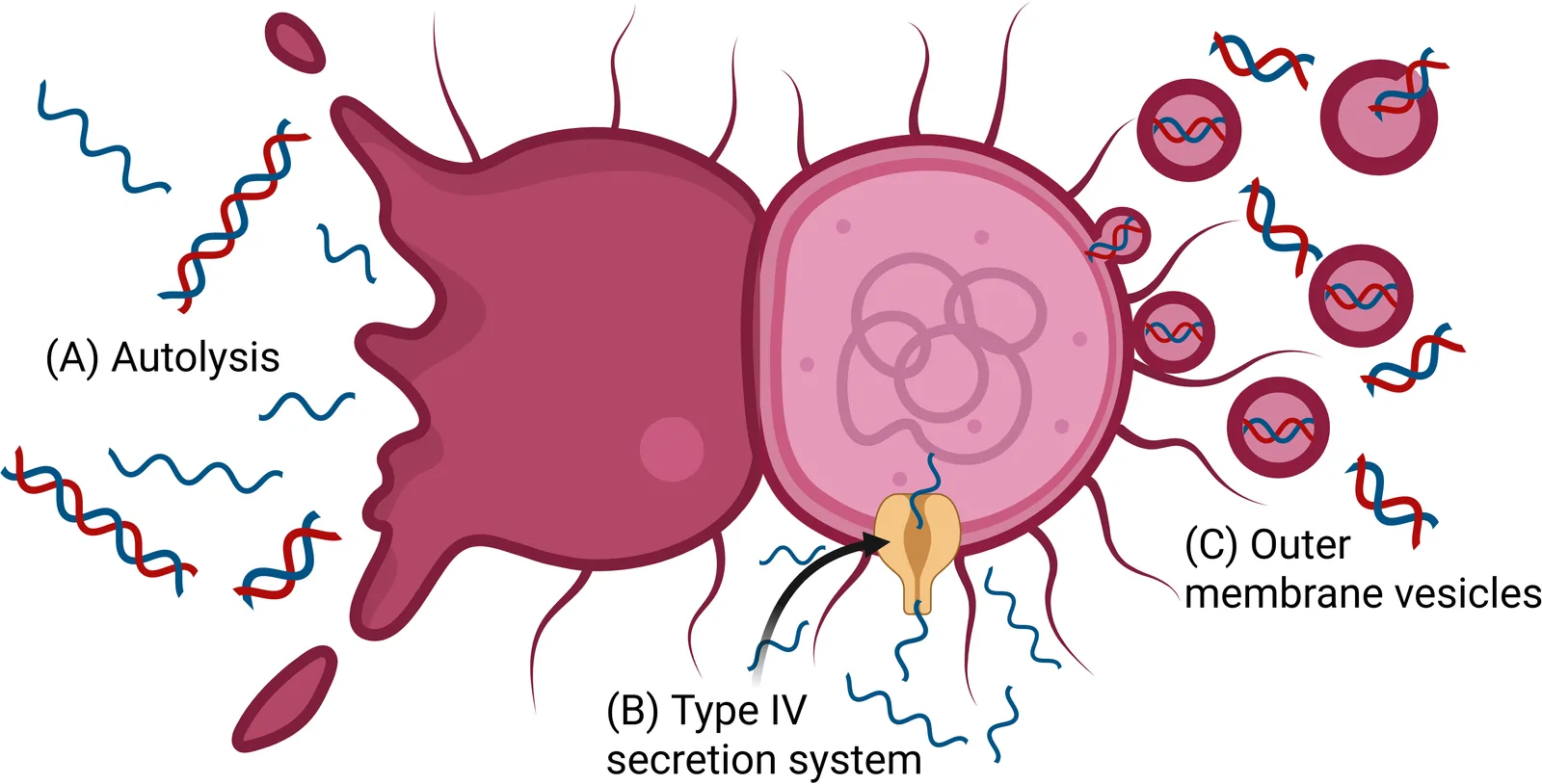
Potential sources of self-derived extracellular DNA in *Neisseria* biofilms. (**A**) Autolysis releasing genomic DNA. (**B**) Secretion of single-stranded DNA via the type IV secretion system. (**C**) Release of double-stranded DNA via outer membrane vesicles. Created in https://BioRender.com.

The most widely recognized source of eDNA in *Neisseria* biofilms is genomic DNA released through autolysis (Fig. 2A) (88, 89). In *N. gonorrhoeae*, autolysis is associated with a prolonged cell division process and is most common when growth stops due to energy depletion or environmental stressors, a process which is mediated by bacteriocin-like peptides (89). By contrast, *N. meningitidis* undergoes less autolysis, and this predominantly occurs in pathogenic meningococcal strains where it releases endotoxin and other virulence factors as well as DNA. It is hypothesized that when growth stops, new peptidoglycan synthesis of these pathogenic *N. meningitidis* clonal complexes also stops, while peptidoglycan degradation via the N-acetylmuramyl-L-alanine-amidase, AmiC, and lytic transglycosylase, LtgA, continues, generating gaps in the cell wall for DNA release during early biofilm formation (63, 68, 90). The autolytic activity of the outer membrane phospholipase A is also important for eDNA release for mechanical stabilization in mature biofilms (68, 91).

Autolysis is most common in meningococcal clonal complexes (i.e., “settler” phenotype) and less frequent in those with the “spreader” phenotype (i.e., ST-11 and ST-8) (45, 68). Both ST-8 and ST-11 clonal complexes are underrepresented in healthy carriers and lack a functional gene encoding the outer membrane phospholipase A for lysis, thus forming eDNA-independent biofilms instead (45, 63, 68, 91).

Commensal *Neisseria* also exhibit autolysis less frequently than *N. gonorrhoeae*. Although early work on *Neisseria pharyngitis* (now reclassified as *Neisseria mucosa*) did not detect autolysis (92), more recent findings indicate that the release of “killer” DNA by *N. elongata.* which can kill both *N. meningitidis* and *N. gonorrhoeae.* appears to be autolytic, based on the autolytic proteins encoded in the *N. elongata* genome (16). The toxicity of this eDNA appears to be due to differences in the methylation pattern rather than its specific genetic content and requires that the DNA is internalized and recombined by the *N. meningitidis* and *N. gonorrhoeae* cells for killing to occur (16).

A second method for DNA release into the ECM is active secretion, which may have evolved as an alternative to autolysis, which is less likely to trigger the host immune system (93). The best-characterized example is in *N. gonorrhoeae*, which secretes single-stranded chromosomal DNA directly into the extracellular space using its type IV secretion system (T4SS) (Fig. 2B) (94, 95). The T4SS complex is encoded on a 59-kb gonococcal genetic island (GGI) and was the first genetic locus responsible for DNA secretion to be characterized (94–96). The single-stranded DNA released by the T4SS complex is blocked at the 5′ end, meaning that only its 3′ end is susceptible to degradation by single-stranded DNA-specific nucleases (93, 95). Although shown to be important during early biofilm formation, this single-stranded DNA is not retained in mature gonococcal biofilms (75). There are 21 proteins that are required for T4SS-mediated eDNA release, including those that make up the complex (96, 97). Two of these include the lytic transglycosylase, AtlA, which is likely involved in hydrolyzing the peptidoglycan layer without inducing lysis, and the partitioning proteins, ParA and ParB, which likely guide the DNA-to-be-secreted to the T4SS complex, both of which are also encoded on the GGI (94, 97–99). However, AtlA and ParB are not present in every version of the GGI, with some gonococcal AtlA being replaced by a different peptidoglycan degradation protein, EppA or some GGI having a ParB deletion (along with other genes), rendering them unable to secrete DNA (97, 100, 101).

The GGI encoding the T4SS is integrated into the chromosomal replication terminus of the gonococcal chromosome, with half of it encoding genes related to the T4SS machinery, while the other half encodes hypothetical proteins, some of which are related to DNA-binding or DNA-processing (96). Approximately 80% of *N. gonorrhoeae* clinical isolates carry the GGI (96). In contrast, only ~18% of sequenced *N. meningitidis* clinical isolates carry the GGI, and where this is present, the genes encoding the T4SS are disrupted by insertions and deletions, which likely renders the complex nonfunctional and incapable of exporting eDNA (100). Although the GGI has also been found in the genome of the commensal *Neisseria bacilliformis*, none of the 15 commensal *Neisseria* spp. (including *N. cinerea*, *N. lactamica,* and *Neisseria mucosa*) encode the *atlA* or *parB* genes needed for single-stranded DNA release (97). Therefore, it appears that single-stranded DNA secretion is specific to *N. gonorrhoeae,* potentially as an adaptation to its niche in the reproductive tract and not a common feature of other *Neisseria* spp. (94).

Finally, eDNA in *Neisseria* spp. may be exported via outer membrane vesicles, a process closely associated with active secretion, albeit less tightly regulated (Fig. 2C) (102). These outer membrane vesicles or blebs were found to harbor large quantities of DNA and DNA-binding proteins in *Neisseria* biofilms (103). Outer membrane vesicles are also abundant in *N. meningitidis* and other commensal *Neisseria* like *N. cinerea*, suggesting a universal mode of eDNA transport across the *Neisseria* genus (104, 105). This mode of transport may also be common in a variety of other bacteria that produce these vesicles, including *Acinetobacter baumannii*, *Helicobacter pylori,* and *Pseudomonas aeruginosa* (106–108). Investigations into the DNA cargo of these vesicles in other bacteria show strong similarity to genomic DNA with wide distribution around the ECM and arrangement in a structured network (109). The packaging of DNA into these vesicles is proposed to occur either as a spontaneous side-effect or consequence of cell lysis or as a result of cell division (110). The type of DNA transported in these outer membrane vesicles and the stage of growth for activation are still unknown for members of the genus *Neisseria*.

## PROTEINS INVOLVED IN NEISSERIA BIOFILM DYNAMICS

In addition to the T4SS described above, other proteins are involved establishing and organizing neisserial biofilms, as well remodeling and stabilizing the DNA fraction. This was demonstrated in experiments showing that crude chromosomal DNA, but not purified protein-free DNA, can promote biofilm formation in some *Neisseria* (111). Some of these DNA-interacting proteins include the DNA-binding lipoprotein antigen, NhbA, and the IgA protease that are both surface-exposed in *N. meningitidis* (111). Cleavage of these proteins by the autotransporter protease, NalP, releases positively charged peptides into the matrix, which limits exDNA-binding and exDNA-dependent biofilms from forming.

One of the major protein complexes required for biofilm formation is the type IV pilus (T4P), a surface-exposed structure that facilitates cell-to-surface interactions in *Neisseria*, as well as enabling cell-cell interactions that promote cellular aggregation and microcolony formation (112, 113). Post-translational modification of the T4P in *N. gonorrhoeae* by phase and antigenic variation may affect the rupture forces necessary to interrupt cell-cell interactions (70, 114–117). This, in turn, triggers bacterial cell sorting or spatial reorganization into clusters in early biofilms as cells tend to move in the direction of the highest rupture force where they can be pulled the strongest (70, 118). Non-piliated *N. gonorrhoeae* with a low rupture force tend to move toward the expanding front or the boundary of an expanding colony, where they would have a stronger competitive advantage (i.e., optimal space and nutrient availability), as well as a higher chance of dispersal to a new biofilm attachment site (70, 114, 115).

Similarly, microcolony formation in *N. meningitidis* has been shown to be reliant on the twitching motility mediated by T4P (PilT), driven via pilus retraction, and if disrupted, can lead to flat, unstructured biofilms that are unable to colonize vascular networks as efficiently (42, 119–121). However, the role of T4P in meningococcal biofilms is not directly related to its organization, and cells that lack this twitching motion are still able to form biofilms. Rather, it is the microcolony formation, bacterial aggregation, and subsequent formation of a three-dimensional architecture that will be impacted (42, 44). Further, it has been demonstrated, by manipulation of the mean number of exposed pili, that different levels of piliation are required for different processes in *N. meningitidis,* with aggregation and adhesion for biofilm formation requiring >40% piliation (122), which stresses the importance of the T4P for the structure and organization of *Neisseria* biofilms.

Remodeling of the ECM or escape of cells from an established biofilm to colonize other areas is an important part of the biofilm lifecycle of bacteria (65). Extracellular thermonucleases with this role have been identified in a variety of bacteria including *S. aureus*, *Vibrio cholerae,* and *B. subtilis,* where their disruption leads to the formation of thicker biofilms and larger cell aggregates with lower rates of detachment (79, 123–125). These extracellular nucleases likely possess a dual role in remodeling the three-dimensional structure of biofilms via eDNA manipulation, as well as the dispersal and detachment of mature biofilms, leading to the availability of eDNA for other processes like nutrient recycling. In *N. gonorrhoeae,* the extracellular Nuc nuclease is homologous to the secreted thermonuclease from *S. aureus* and is implicated in the development of biofilms, with its deletion leading to the formation of significantly thicker and larger gonococcal biofilm biomass (41). This was directly attributed to an increased level of eDNA via microscopy, which suggested that Nuc may be acting on eDNA by processing it into fragments that are optimal for biofilm formation (41). Apart from directly hydrolyzing eDNA, Nuc is also capable of degrading neutrophil extracellular traps (NETs) in *N. gonorrhoeae*, which then leads to DNA release from the NETs into the ECM, potentially offsetting the digestion of biofilm eDNA (126). Although little is known about the presence of external nucleases in other *Neisseria* spp., homologs of Nuc are present in the genome of *N. meningitidis* and many commensal *Neisseria* like *N. lactamica* and *N. cinerea*, highlighting its potential role in biofilm formation across the *Neisseria* genus.

In addition to this, our group has recently shown that the disruption of the ATP-dependent DNA ligase LigE in *N. gonorrhoeae* reduces biofilm formation (127, 128). LigE is also found in many other bacteria where eDNA seems to play a central role in biofilm formation including *Campylobacter jejuni*, *Haemophilus influenzae*, *N. meningitidis,* and *V. cholerae* (129). Considering the presence of a periplasmic localization signal peptide on the N-terminus of LigE, we hypothesize that this ligase works outside the cytoplasm to enhance the amount of high-molecular-weight eDNA that enhances biofilm formation, potentially counteracting the effects of the Nuc thermonuclease.

## FUTURE DIRECTIONS: TARGETING PATHOGENS AND UNDERSTANDING COMMENSALS

Our increased understanding of both the functions of eDNA in biofilm and its sources offers a possible pathway to target *N. meningitidis* and *N. gonorrhoeae* infection in the face of increasing antibiotic resistance. The potential use of nucleases to disperse these biofilms could combat their persistence during infection and aid the effectiveness of antibiotic therapies and the host immune response. Nucleases provided early research tools in the study of DNA as a component of bacterial biofilm (130–132) and in the case of *P. aeruginosa* have been developed into an aerosolized formulation of recombinant human DNaseI used as a therapeutic Pulmozyme (dornase alfa) for cystic fibrosis patients (133). Similar to the early experiments with *P. aeruginosa*, DNase inhibition of *N. gonorrhoeae* biofilm formation was crucial in establishing eDNA as a major component of gonococcal biofilms (41, 50). This suggests a nuclease-based strategy could be beneficial in disrupting biofilms, which could make systemic treatment more effective. Although systemic antibiotic treatment will likely remain necessary, mechanical or biochemical disruption of biofilm would increase antibiotic penetration of bacterial colonies and potentially slow evolution of resistance by ensuring a therapeutic concentration is experienced consistently across the population. However, it is also necessary to consider that biofilm disruption could increase the spread or transmission of pathogenic *Neisseria*, based on the role of stable biofilm in the settler/spreader model of transmission. Such a potential increase in pathogen spread could counteract the possible positive outcomes of decreasing biofilm, and both would need to be experimentally evaluated.

The evidence that eDNA can be both a source of transforming DNA, potentially disseminating resistance and virulence genes among pathogens, and a bactericidal agent in inter-species competition recommends future research into its functional roles in biofilm. This will likely involve not just characterization of the nucleic acid component of the biofilm but also a detailed understanding of its interaction with proteins at the bacterial cell surface and periplasm, which are involved in DNA export, recognition, and uptake.

In this review, we also draw attention to the paucity in research on biofilm formation by commensals, particularly relative to our understanding of biofilm in their pathogenic counterparts. A more thorough understanding of commensal biofilm in host colonization, the role of *Neisseria* in mixed biofilms of healthy human microbiota, and the role of commensal *Neisseria* in competition with other bacteria would provide a better understanding of the fundamental workings of the genus. Further, a better understanding of the role of biofilm in interspecies interactions could potentially be used to combat pathogenic *Neisseria* carriage and transmission.
